# A Method Sustaining the Bioelectric, Biophysical, and Bioenergetic Function of Cultured Rabbit Atrial Cells

**DOI:** 10.3389/fphys.2017.00584

**Published:** 2017-08-15

**Authors:** Noa Kirschner Peretz, Sofia Segal, Limor Arbel-Ganon, Ronen Ben Jehuda, Yuval Shemer, Binyamin Eisen, Moran Davoodi, Ofer Binah, Yael Yaniv

**Affiliations:** ^1^Biomedical Engineering Faculty, Technion – Israel Institute of Technology Haifa, Israel; ^2^Department of Physiology, Biophysics and Systems Biology, Technion – Israel Institute of Technology Haifa, Israel; ^3^The Rappaport Institute, The Ruth and Bruce Rappaport Faculty of Medicine, Technion – Israel Institute of Technology Haifa, Israel

**Keywords:** atrial fibrillation, biophysics, energetics, mitochondria, sarcoplasmic reticulum

## Abstract

Culturing atrial cells leads to a loss in their ability to be externally paced at physiological rates and to maintain their shape. We aim to develop a culture method that sustains the shape of atrial cells along with their biophysical and bioenergetic properties in response to physiological pacing. We hypothesize that adding 2,3-Butanedione 2-monoxime (BDM), which inhibits contraction during the culture period, will preserve these biophysical and bioenergetic properties. Rabbit atrial cells were maintained in culture for 24 h in a medium enriched with a myofilament contraction inhibitor, BDM. The morphology and volume of the cells, including their ability to contract in response to 1–3 Hz electrical pacing, was maintained at the same level as fresh cells. Importantly, the cells could be successfully infected with a GFP adenovirus. Action potentials, Ca^2+^ transients, and local Ca^2+^ spark parameters were similar in the cultured and in fresh cells. Finally, these cultured cells' flavoprotein autofluorescence was maintained at a constant level in response to electrical pacing, a response similar to that of fresh cells. Thus, eliminating contraction during the culture period preserves the bioelectric, biophysical and bioenergetic properties of rabbit atrial myocytes. This method therefore has the potential to further improve our understanding of energetic and biochemical regulation in the atria.

## Introduction

Cardiac fibrillation is one of the leading causes of morbidity and mortality in the developed world, where atrial fibrillation (AF) is the most common sustained arrhythmia (Benjamin et al., 2017), affecting ~2.2 million patients in the United States, and its prevalence increases with age (one in four people over the age of 40 will develop AF; Lloyd-Jones et al., 2004). Although, AF is not considered a direct life-threatening arrhythmia, it affects the circulatory system of patients and their general health and quality of life; furthermore, AF poses a significant burden on the healthcare system (Kirchhof et al., 2016). For example, AF is a significant risk factor for stroke, with about 15% of strokes occurring in people with AF (Wolf et al., 1991).

Concomitantly to changes in ionic channels and other membrane molecules (Nattel et al., 2007), alterations in Ca^2+^ dynamics (Macquaide et al., 2015), phosphorylation state (Heijman et al., 2013), and energetic balance (Kalifa et al., 2008) have in recent years been associated with AF. Gene manipulation, GFP gene transfection, and Förster resonance energy transfer methods have recently been developed to explore biophysical, bioelectrical and bioenergetic mechanisms (Fischer et al., 2015; Yaniv et al., 2015; Behar et al., 2016; Wüst et al., 2017). Although, these methods are bringing new insights into AF-related molecular mechanisms, they must be applied on cells that are maintained in culture at least for 24 h. Currently, cultured rabbit atrial cells (or atrial cells from any other mammal) cannot be electrically paced at rates higher than 0.5 Hz, whereas the spontaneous beating rate of rabbit sinoatrial node cells is 3 Hz (Rinne et al., 2010; Hohendanner et al., 2015). Moreover, the atrial cell shape changes, and therefore the distances between intercellular compartments are altered (Gilliam et al., 1989). Proper measurements of physiological Ca^2+^ dynamics, posttranslational modification signaling, and energetic balance in cultured cells are thus impossible, and AF-related mechanisms cannot be explored.

Here we report a novel culture method which makes it possible to sustain the bioelectric, biophysical, and bioenergetic functions of atrial cells. Most importantly, the cultured cells can be infected with a GPF adenovirus after 24 h of culture. The essence of this method is the addition of a myofilament contraction inhibitor [2,3-Butanedione 2-monoxime (BDM)] to the culture medium for 24 h. This method therefore has the potential to further improve our understanding of energetic and biochemical regulation in the atria, which can lead to novel therapies involving the adaptation of this signaling, with the goal of eliminating AF events.

## Materials and methods

### Animal use

Animals were treated in accordance with the Technion Ethics Committee. The experimental protocols were approved by the Animal Care and Use Committee of the Technion (Ethics number: IL-118-10-13).

### Atrial cell isolation

Atrial tissue was isolated from healthy New Zealand White rabbits of either sex (70 males and 4 females) weighing 2.3–2.7 kg. Each rabbit was weighed and sedated through administration of an intramuscular injection containing Ketamine (0.1 ml/kg) and Xylazine (0.1 ml/kg). An intravenous (IV) cannula was inserted in the rabbit's ear for the future use of an anesthetic. The rabbit was administered 200 mg/ml sodium pentobarbital diluted with heparin through the IV cannula. The adequacy and efficiency of the anesthesia were examined by observing the loss of reflexes in the eye and foot. Then the rabbit was placed on its back, the skin over the sternum was pulled back, and careful cuts were made through the diaphragm and the ribs, revealing the heart. The heart was removed quickly and placed in a cold PBS buffer without MgCl_2_ and CaCl_2_ (Sigma Aldrich). The heart was placed in a chamber coated with wax (Sigma Aldrich) superfused through a peristatic pump (Masterflex L/s, Cole-Parmer) at a speed of 20–25 ml/min and at a temperature of 37°C with Tyrode's solution that contained (in mM) NaCl 125, KCl 5.6, NaH_2_PO_4_ 1.2, NaHCO_3_ 24, glucose 5.6, MgCl_2_ 1, and CaCl_2_ 1.8, bubbled with 95% O_2_ and 5% CO_2_. The superfusion proceeded throughout the isolation process. The atrial and the sinoatrial tissues are separated by the crista terminalis region, in which their beating is visible. The atrial tissue was separated from the sinoatrial node, cut into small strips, and washed twice (15 min each round) in a Ca^2+^-free isolation solution that contained (in mM) NaCl 140, KCl 5.4, NaH_2_PO_4_ 0.33, HEPES 5, glucose 5.5, MgCl_2_ 1, and taurine 50 (pH 7 with NaOH), bubbled with 100% O_2_ to adjust the pH to 7.4 at 34.6°C and shaken at a rate of 100 RPM (61.5 g). Afterwards, the atrial tissue was incubated at 34.6°C for 40 min in 5 ml of Ca^2+^-free isolation solution that contained an enzyme cocktail (collagenase type II (Worthington), 3 mg; elastase type IV (Sigma Aldrich), 3.4 mg: protease type IV (Sigma Aldrich), 0.4 mg; and bovine serum albumin (BSA) (Sigma Aldrich), 1.8 mg) shaken at a rate of 80 RPM. The tissue was washed twice (10 min each round) at room temperature in a KB solution containing (in mM) L-glutamic Acid 70, KCl 30, KH_2_PO_4_ 10, HEPES 10, taurine 20, glucose 10, MgCl_2_ 1, and EGTA 0.3 (pH 7.38 with KOH). Cells were dispersed from the atrial tissue preparation by gentle pipetting through the KB solution, filtered through a 150 μm mesh, and stored at 4°C to be used fresh for 6 h.

### Culture procedure

The cells were placed in a short term culture (24 h). The cells were seeded onto 35 mm glass dishes (MatTek Corporation, Ashland, MA, U.S.) coated in advance (for 1 h at 37°C, 90% humidity & 5% CO_2_, Galaxy 170R, Eppendorf) with 25 μg/ml laminin (Sigma Aldrich) dissolved in PBS (in 1 × PBS + 1% PS, no Ca^2+^ and no Mg^2+^, Sigma Aldrich). The laminin coating was aspirated at least 10 min prior to seeding the cells. The cells were diluted in a serum-free culture medium which contained M199 (Sigma Aldrich), 2% PS [penicillin-streptomycin (Gibco)], 1% ITS (insulin-transferrin-selenium, Sigma Aldrich), 0.1% BDM (2, 3 butanedione, Sigma Aldrich), and 0.01% BSA (Sigma Aldrich) and were then centrifuged at 1,000 RPM for 10 min (Spectrafuge 6C, Labnet). The conical tube containing the cells was drained, leaving the cells at the bottom of the tube. 600 μl of the serum-free medium was added to the conical tube and the cell suspension was divided between 3 dishes. The dishes were incubated for 1 h (for 1 h at 37°C, 90% humidity & 5% CO_2_, Galaxy 170R, Eppendorf). The cells were washed three times with a serum-enriched medium that contained M199, 5% FBS (fetal bovine serum, Gibco), 2% PS and 0.1% BDM, and 2 ml of the serum-enriched medium was added to each dish. The cells were placed in incubation for 24 h prior to further experiments.

### Experimental medium

During live microscopy measurements the cells were washed with a HEPES buffer containing (in mM): NaCl 140, KCl 5.4, HEPES 5, Glucose 10, MgCl_2_ 2, CaCl_2_ 1 (pH 7.4 with NaOH).

### Quantifying cell length, width volume and sarcomere length

To quantify atrial cell shape, the cells were imaged on an inverted fluorescence microscope (Zeiss Observer Z1, Germany) using a 40x/1.4 N.A oil immersion lens. The atrial cells were placed in a 35 mm round glass dish (MatTek Corporation, Ashland, MA, U.S.) with HEPES solution (see above) inside a microscope incubator (Zeiss, Germany) at 37 ± 0.5°C. Cells were allowed to settle for 10 min and were imaged under Z-stack mode (slice = 0.99 μm, 30–50 frames per image). After microscopic recording, cell properties (length, width and volume) were measured with the ZEN 2 edition from Carl Zeiss Microscopy (GmbH software, Germany). To calculate the volume, for each cell, a cross-section area was taken by marking the perimeter manually, and the depth was taken from the microscope's Z-stack parameters. To calculate the sarcomere length, a clear line of sarcomeres was chosen, Fourier transform was done on its intensity, and the sarcomere length was calculated from the dominant peak.

### Electrical stimulation

Global and local Ca^2+^ release, flavoprotein autofluorescence, and action potential were measured during electrical field stimulation (1, 2, and 3 Hz) using a pair of platinum electrodes (0.008″ bare wire, A-M Systems) glued to a custom-made chamber top.

### Electrophysiology

APs were recorded as described previously (Ben-Ari et al., 2016). The atrial cells were plated on glass coverslips (13 mm diameter). In short, APs were recorded in whole-cell patch- clamp mode. The patch pipette solution contained (mmol/l) 120 KCl, 1 MgCl_2_, 3 Mg-ATP, 10 HEPES, and 10 EGTA titrated to pH 7.2 with KOH and adjusted at 290 mOsm with saccharose (all materials were purchased from Sigma-Aldrich). APs were recorded for data amplification, acquisition, and analysis using an Axopatch 200B, Digidata 1322 and pClamp10 (Molecular Devices, Sunnyvale, CA, US). Signals were digitized at 10 kHz. Patch electrodes with resistances of 4–7 MΩ were pulled from borosilicate glass capillaries (Harvard Apparatus, MA, US).

### T-tubule visualization

T-tubules were visualized using a potential-sensitive dye Di-8-ANEPPS (1:200 (10 μM), ThermoFisher Scientific). Atrial cells were loaded with 10 μM Di-8-ANEPPS for 10 min at room temperature and were subsequently washed with HEPES solution at 37 ± 0.5°C. The Ca^2+^ fluorescence was imaged by a LSM880 confocal microscope (Zeiss) using a 40x/1.2 water immersion lens. Cells were excited with a 488 nm argon laser. Fluorescence emission was collected with LP 505 nm. All images were captured in frame mode (4,096 × 4,096 pixels at 212.55 × 212.55 μm). The fluorescence of the cell was measured as an index of t-tubule density.

### Immunofluorescence and immunolabeling of atrial myocytes

The freshly isolated cells were allowed to settle for 20 min at room temperature prior to fixation and the cultured cells were fixated ~24 h after seeding. The cells were fixated with 2% formaldehyde (Electron Microscopy Sciences) in PBS (Sigma Aldrich) for 10 min, rinsed with 0.1 Triton X-100/PBS (Sigma Aldrich), and permeabilized with 1% Triton X-100/PBS for 15 min. The cells were placed in blocking solution [PBS, 2% IgG-free BSA (Jackson ImmunoResearch, West Grove, PA, USA), 5% donkey serum (Jackson ImmunoResearch), 0.02% NaN_3_ (Sigma Aldrich), 0.1% Triton (Sigma Aldrich)] for 4 h in order to block nonspecific cross-reactivity, and then they were incubated overnight at 4°C with primary antibodies (see below) diluted in blocking solution. The cells were rinsed with 1% IgG-free BSA/PBS, and conjugated secondary antibodies (1:500, Jackson ImmunoResearch) were applied for 1 h prior to final rinse with 1% IgG-free BSA/PBS. The cells were imaged by a LSM880 confocal microscope (Zeiss, Germany) at room temperature using a 40x/1.2 water immersion lens. Cells were excited with a 488 nm argon laser line, fluorescence emission was collected with LP 505 nm, and all images were captured in frame mode (4,096 × 4,096 pixels at 106.27 × 106.27 μm). The distance between different structures was calculated using the ZEN 2 edition from Carl Zeiss Microscopy (GmbH software, Germany).

### Antibodies

We employed either monoclonal anti-SERCA2 ATPase antibodies (IgG1, clone IID8, 1:500, ThermoFisher Scientific) or monoclonal anti-RyR2 antibodies (IgG1, clone C3-33, 1:100, ThermoFisher Scientific, Bothell, WA, US).

### Ca^2+^ measurements

Ca^2+^ cycling into and out of the cytosol was measured with Fluo-4 AM (ThermoFisher Scientific). Atrial cells were loaded with 5 μM Fluo-4 AM for 20 min at room temperature and were subsequently washed with HEPES solution at 37 ± 0.5°C. The Ca^2+^ fluorescence was imaged by a LSM880 confocal microscope (Zeiss) using a 40x/1.2 water immersion lens. Cells were excited with a 488 nm argon laser. Fluorescence emission was collected with LP 505 nm with the pinhole set to form an image of no more than a 3 μm optical slice (512 × 1 pixels at 106.07 pixels/μm and 4.94 ms/line for a 1 Hz stimulation rate, at 4.07 ms/line and 106.07 pixels/μm for a 2 Hz stimulation rate and 3.2 ms/line and 106.07 pixels/μm for a 3 Hz stimulation rate). All images were recorded with a line scan function oriented to scan along the long axis of the cell, close to the sarcolemmal membrane. Ca^2+^ images were analyzed on a custom-made GUI programed in Matlab (Davoodi et al., 2017). The Ca^2+^ image was normalized to the background fluorescence.

### Mitochondrial membrane potential measurements

Mitochondrial membrane potential was visualized using a potential-sensitive dye TMRM (1:80 (125 nM), tetramethylrhodamine methyl ester perchlorate, ThermoFisher Scientific). The cells were imaged by a LSM880 confocal microscope (Zeiss) at room temperature using a 40x/1.2 water immersion lens. Cells were excited with a 543 nm argon laser, and fluorescence emission was collected with LP 567 nm. All images were captured in frame mode (512 × 512 pixels at 212.55 × 212.55 μm).

### Flavoprotein autofluorescence

In order to properly define the bioenergetic state of the atrial myocytes, the autofluorescence of mitochondrial flavoprotein was imaged at different electrical stimulation frequencies (quiescent, 1, 2, and 3 Hz) at 37 ± 0.5°C by an inverted fluorescence microscope (Zeiss Observer Z1) using a 40x/1.4 N.A oil immersion lens and a 445 nm LED. Images were recorded at a rate of 2 frames per second. The flavoprotein fluorescence was normalized to the level measured during quiescent mode per cell.

### Infection of cultured atrial myocytes

Cells were infected with adenovirus (1:100) expressing green fluorescent protein (GFP). The cells were infected with the virus 1 h after seeding and were imaged 24 h after infection. To evaluate the infection, the cells were imaged on an inverted fluorescence microscope (Zeiss Observer Z1) at 37 ± 0.5°C using a 40x/1.4 N.A oil immersion lens and 488 nm LED excitation. All images were captured in frame mode (448 × 500 pixels at 152.54 × 170.25 μm).

### Statistics

Data are presented as mean ± SEM. A paired *t*-test was employed to compare the means of paired samples. For independent samples, a two-sample *t*-test was applied. *P* < 0.05 was taken to indicate statistical significance.

## Results

### Cultured cells preserve their shape and volume similarly to fresh cells

**Figure 1:**
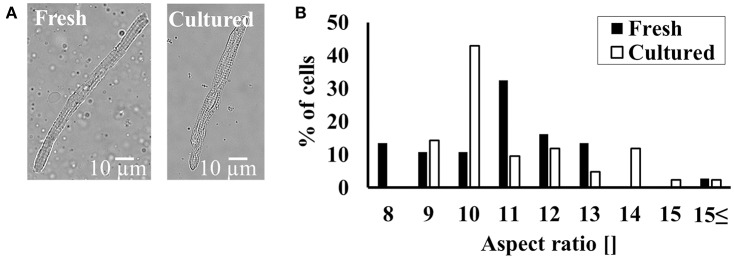
Maintaining cultured cell volume and shape. **(A)** Representative example of fresh and cultured atrial cells. **(B)** Distribution of cell aspect ratio before (*n* = 38; cells taken from 18 rabbits) and in culture with BDM (*n* = 43; cells taken from 18 rabbits).

Cell shape determines the distance between internal structures and as such is vital to internal communication and atrial function. Figure 1A shows a representative example of fresh and cultured atrial cells. On average, the cells maintained their rectangular shape in the presence of BDM. Moreover, on average, fresh cells have similar length (101 ± 4 μm; *n* = 38; cells were taken from 18 rabbits) as to cultured cells (105 ± 4 μm; *n* = 43; cells were taken from 18 rabbits). Likewise, fresh cells have similar width (9.8 ± 0.3 μm; *n* = 38; cells were taken from 18 rabbits) as to cultured cells (10.1 ± 0.4 μm; *n* = 43; cells were taken from 18 rabbits). The distribution around the mean of the aspect ratio (Figure 1B) does not differ significantly between cultured and fresh cells (quantified by a Kolmogorov-Smirnov two-sample test). Moreover, the sarcomere length did not differ between fresh cells (1.98 ± 0.08; *n* = 36 from 18 rabbits) and cultured cells (1.94 ± 0.08; *n* = 43; cells were taken from 18 rabbits).

### Cultured cells can be externally paced at their physiological rate

**Figure 2:**
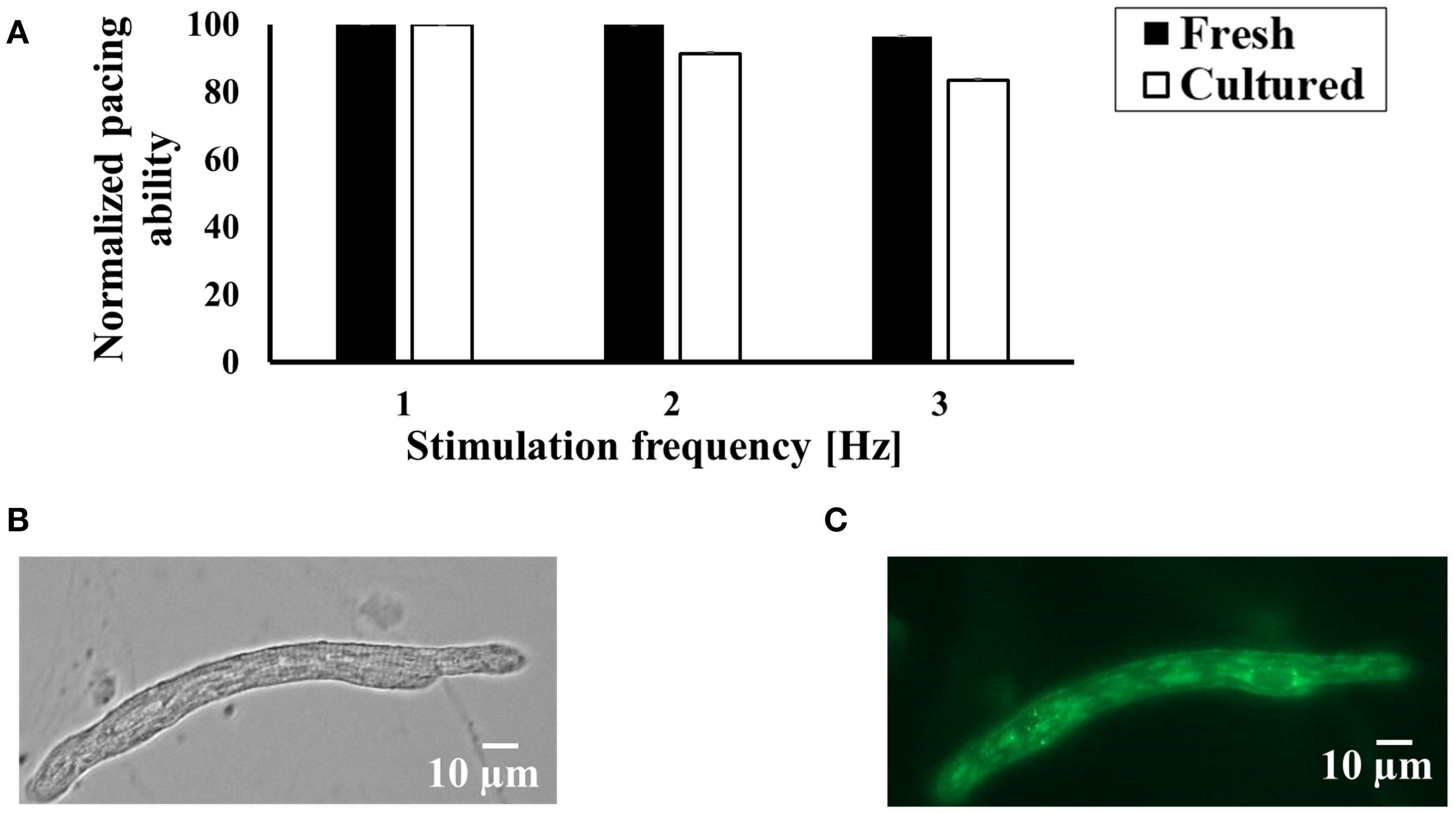
The ability of cultured cells to contract in response to external electrical stimulation and express exogenous proteins. **(A)** Average success rate for electrically paced fresh and cultured cells (*n* = 144 fresh cells from 22 rabbits; *n* = 51 cultured cells from 22 rabbits) at a rate of 1, 2, and 3. The results are normalized to the number of cells successfully paced at 1 Hz. **(B)** Bright field and **(C)** fluorescence images of representative atrial cells 24 h after infection with adenoviruses encoding eGFP (Ad-GFP).

In order to measure bioelectric, biophysical and bioenergetic properties of atrial cells, they must be responsive to external pacing at their physiological rate [the spontaneous beating rate of a rabbit pacemaker cell is around 3 Hz (Lyashkov et al., 2007)]. Figure 2A shows that in response to increased electrical pacing (1, 2, and 3 Hz), the cultured cells responded similarly to fresh cells, indicating that the cell culture protocol preserves the ability of the cells to be paced. Note that because not all the fresh and cultured cells can be paced, we chose only cells that can be paced at least at 1 Hz (far from the physiological rate). Thus, our control level is 1 Hz of stimulation. On average, we succeeded to pace 37% of fresh cells and 37.5% in culture.

### Cultured cells can be infected with adenovirus

Because our central goal was to establish an experimental system in which exogenous proteins can be introduced into cultured atrial cells, we transfected the cells for 24 h with adenovirus delivering cDNA encoding eGFP. Figures 2B,C show a representative cell that was infected with adenovirus. We obtained nearly 100% infection efficiency with no toxicity or change in cell morphology. Cell area was measured to be 739 ± 185 μm^2^ (*n* = 8; cells were taken from 3 rabbits) and 900 ± 80 μm^2^ (*n* = 66; cells were taken from 3 rabbits), respectively, in cultured cells with and without the virus (*p* = 0.27).

### Eliminating BDM from the culture resulted in the loss of ability to maintain cell volume and in the ability of the cells to be externally paced

**Figure 3:**
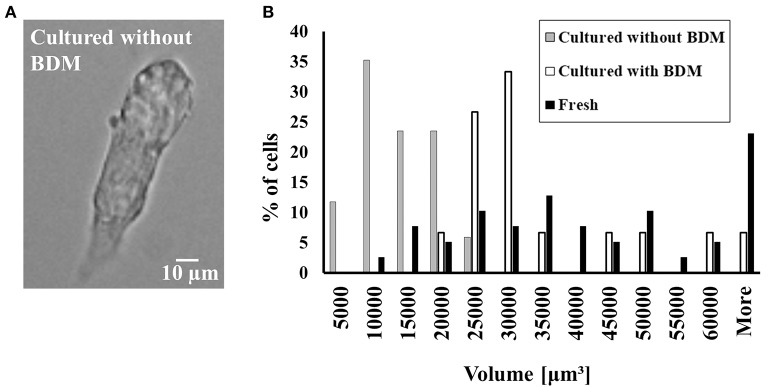
The effect of BDM on cultured cell volume and shape and the ability of cultured cells to contract in response to external electrical stimulation. **(A)** Representative example of cultured atrial cell without BDM. **(B)** Distribution of cell volume before (*n* = 38; cells taken from 22 rabbits) and in culture with (*n* = 43; cells taken from 22 rabbits) and without (*n* = 17; cells taken from 1 rabbit) BDM.

To prove that the presence of BDM is the main factor in the preservation of cell volume, we cultured cells in the absence and presence of BDM and compared them to fresh cells. Figure 3A shows a representative cell that could not maintain its shape in the absence of BDM in the cultured medium. Because atrial cells cultured without BDM do not maintain their rectangular shape, we quantified volume and not length and width. Figure 3B shows that in the absence of BDM, the cultured atrial cells do not maintain their volume compared to cultured cells with BDM (quantified by a Kolmogorov-Smirnov two-sample test). Moreover, we could not pace the cells cultured without BDM at any stimulation rate (*n* = 17 from 3 rabbits); thus, these cells could not generate action potentials (APs) or Ca^2+^ transients; nor could we test their ATP supply to demand balance in response to electrical pacing. Therefore, we did not continue to use this medium for other experiments.

### AP characteristics in cultured cells

**Figure 4:**
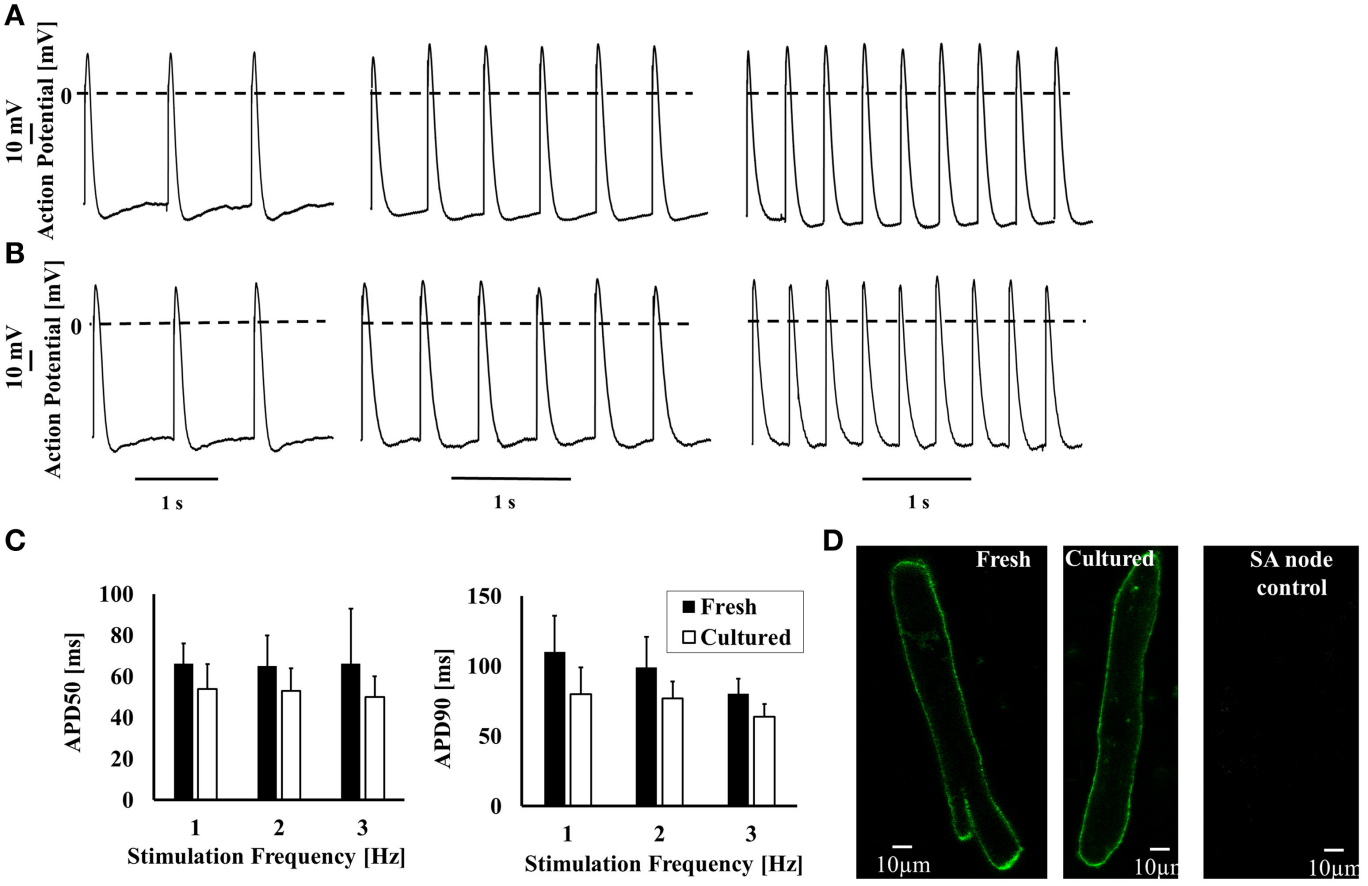
The ability of cultured cells to generate AP in response to external electrical stimulation. Representative examples of **(A)** fresh and **(B)** cultured AP in atrial cells at a stimulation rate of 1, 2, and 3. **(C)** Average APD_50_ and APD_90_ for fresh and cultured cells. **(D)** Representative examples of fresh and cultured atrial cells loaded with Di-8-ANEPPS to visualize t-tubules.

Next, we determined whether APs of cultured cells respond to electrical pacing similarly to fresh cells. Figures 4A,B show representative APs recorded from fresh and cultured cells, respectively. Table 1 and Figure 4C shows that maximal diastolic potential (MDP), AP amplitude, and action potential duration at 50 and 90% repolarization (APD_50_ and APD_90_, respectively) do not differ significantly between fresh and cultured cells. Note that only cells that can be externally paced were measured for electrophysiology, Ca^2+^ characteristics and bioenergetics signals. Moreover, no current was injected to lower the potential to evoke AP. We determined whether t-tubules were lost during culture. Figure 4D shows t-tubule staining in both cultured and fresh atrial cells. Analysis of average intensity of Di-8-ANEPPS (see Materials and Methods for further details) shows that this index does not change between fresh and cultured cells (*p* = 0.2): 10.6 ± 1 (*n* = 21) vs. 9.6 ± 1 (*n* = 38), respectively. Thus, although t-tubule density is lower in atrial cells than in ventricular, it is similar in fresh and cultured cells.

**Table 1:** Action potential (AP) parameters.

	**Fresh 1Hz**	**Cultured 1Hz**	**Fresh 2Hz**	**Cultured 2Hz**	**Fresh 3Hz**	**Cultured 3Hz**
Amplitude [mV]	89 ± 4	84 ± 12	93 ± 6	90 ± 13	98 ± 12	96 ± 12
		*p =* 0.2		*p =* 0.2		*p =* 0.23
Overshoot [mV]	31 ± 2	30 ± 9	33 ± 5	34 ± 9	38 ± 9	41 ± 9
		*p =* 0.3		*p =* 0.08		*p =* 0.2
APD_50_ [ms]	66 ± 10	54 ± 12	65 ± 15	53 ± 11	66 ± 27	50 ± 10
		*p =* 0.08		*p =* 0.1		*p =* 0.09
APD_90_ [ms]	110 ± 26	80 ± 19	99 ± 22	77 ± 12	80 ± 11	64 ± 9
		*p =* 0.07		*p =* 0.09		*p =* 0.08
Maximal diastolic potential	−58 ± 4	−57 ± 5	−60 ± 3	−59 ± 5	−60 ± 3	−57 ± 5
[mV]		*p =* 0.3		*p =* 0.4		*p =* 0.6
Number of cells	5	6	5	6	5	6
Number of rabbits	5	5	5	5	5	5

### Ca^2+^ transient and local Ca^2+^ release characteristics in cultured cells

**Figure 5:**
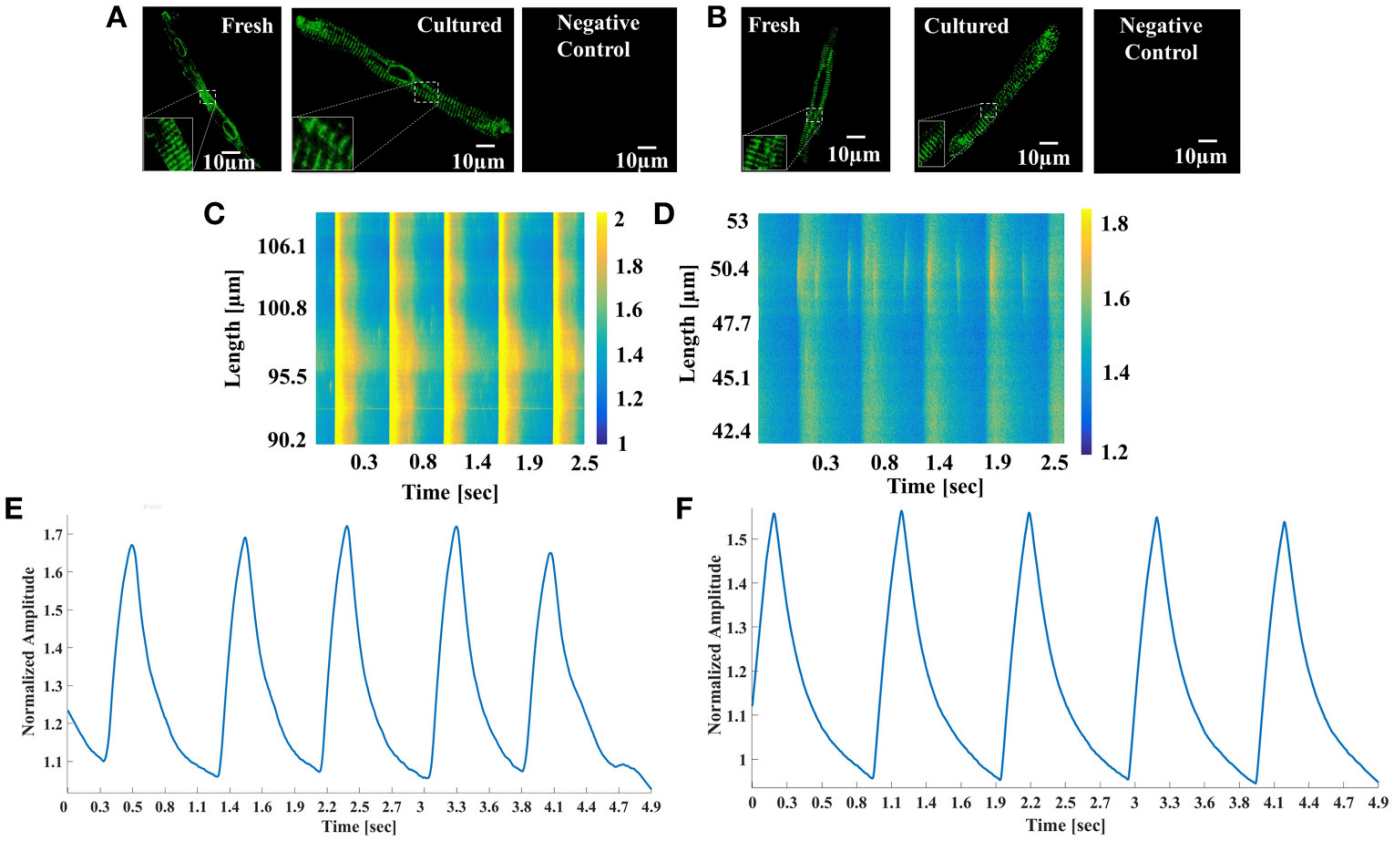
SERCA and Ryanodine structure and Ca^2+^ signal. Representative examples of **(A)** SERCA and **(B)** ryanodine immunolabeling in fresh and cultured cells. Representative examples of Ca^2+^ imaging in **(C)** fresh and **(D)** cultured atrial cells. Representative examples of Ca^2+^ transients in **(E)** fresh and **(F)** cultured atrial cells.

**Figure 6:**
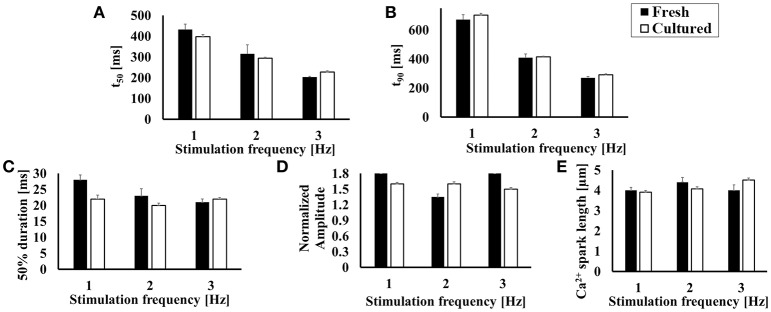
Global and local Ca^2+^ spark characteristics. **(A)** 50% and **(B)** 90% relaxation of Ca^2+^ transient (*n* > 6; for exact number for each frequency, see Table 2). Local Ca^2+^ spark characteristics: **(C)** 50% duration, **(D)** normalized amplitude, and **(E)** length (*n* > 41; for exact number for each frequency, see Table 2).

In atrial cells Ca^2+^ signaling is vital for cell homeostasis, contraction, and energy balance. Because cultured atrial cells can be electrically paced, we quantified Ca^2+^ transients and local Ca^2+^ release characteristics using line scan in cultured cells compared to fresh cells. First, we confirmed that ryanodine receptors (RyRs) and SERCA remain intact in the cultured cells. Figures 5A,B show immunolabeling of SERCA and RyR in fresh and cultured cells, respectively. Both images illustrate high density with a repetitive pattern of SERCA and RyR distribution in both cell types. Indeed, the distance between RyR (1,556 ± 92 (*n* = 5) and 1,760 ± 20 nm (*n* = 10), *p* = 0.1 for fresh and cultured cell, respectively) and SERCA (1,780 ± 24 (*n* = 4) and 1,760 ± 12 nm (*n* = 10), *p* = 0.94 for fresh and cultured cells, respectively) was similar. After ascertaining that the structures responsible for the Ca^2+^ flux from the SR was unaltered, we quantified the Ca^2+^ release kinetics. Figures 5C–F show representative examples of Ca^2+^ imaging and Ca^2+^ transients in fresh and cultured cells, respectively. Figures 6A,B show that mean 50 and 90% relaxation times do not differ significantly between fresh and cultured cells. Similarly, the comparison of local Ca^2+^ release characteristics in Figures 6C–E show no significant difference between fresh and cultured cells with regard to 50% duration, amplitude, and Ca^2+^ spark length. For additional Ca^2+^ transient and local Ca^2+^ release characteristics, see Table 2. Note that none of these characteristics differ significantly between fresh and cultured cells.

**Table 2:** Ca^2+^ cycling and spark parameters.

**Parameter name**	**Fresh 1Hz**	**Cultue 1Hz**	**Fresh 2Hz**	**Cultur 2Hz**	**Fresh 3Hz**	**Culture 3Hz**
Time to peak [ms]	276 ± 20	239 ± 7	208 ± 16	184 ± 3	130 ± 11	145 ± 2
		*p =* 0.08		*p =* 0.09		*p =* 0.1
t_50_ [ms]	418 ± 26	412 ± 17	327 ± 24	293 ± 5	212 ± 7	227 ± 6
		*p =* 0.09		*p =* 0.08		*p =* 0.4
t_90_ [ms]	653 ± 37	714 ± 16	437 ± 19	416 ± 5	277 ± 8	295 ± 5
		*p =* 0.16		*p =* 0.1		*p =* 0.2
Cycle length [ms]	983 ± 33	999 ± 3.6	508 ± 1.7	508 ± 2	336 ± 8	342 ± 4
		*p =* 0.2		*p =* 1		*p =* 0.5
Ca^2+^ spark 50% duration [ms]	28 ± 1.5	22 ± 1.1	22 ± 1.5	20 ± 0.8	20 ± 0.9	21 ± 0.8
		*p =* 0.1		*p =* 0.24		*p =* 0.18
Ca^2+^ spark amplitude []	1.8 ± 0.1	1.6 ± 0.03	1.3 ± 0.04	1.6 ± 0.04	1.7 ± 0.08	1.5 ± 0.03
		*p =* 0.09		*p =* 0.09		*p =* 0.09
Ca^2+^ spark length [μm]	4 ± 0.1	3.9 ± 0.1	4.2 ± 0.18	4.07 ± 0.1	4.06 ± 0.2	4.6 ± 0.1
		*p =* 0.16		*p =* 0.26		*p =* 0.09
Time between Ca^2+^ spark and former Ca^2+^ peak [ms]	562 ± 21	518 ± 30	334 ± 22	276 ± 12	169 ± 18	180 ± 8
		*p =* 0.2		*p =* 0.1		*p =* 0.1
Spark/μm/sec	0.03 ± 0.01	0.024 ± 0.001	0.02 ± 0.001	0.02 ± 0.002	0.02 ± 0.001	0.03 ± 0.001
		*p =* 0.2		*p =* 0.3		*p =* 0.09
Number of cells	8	20	9	23	6	20
Number of rabbits	6	3	6	3	6	3
Number of Ca^2+^ sparks	122	144	69	223	41	179

### Bioenergetic signaling in cultured cells

**Figure 7:**
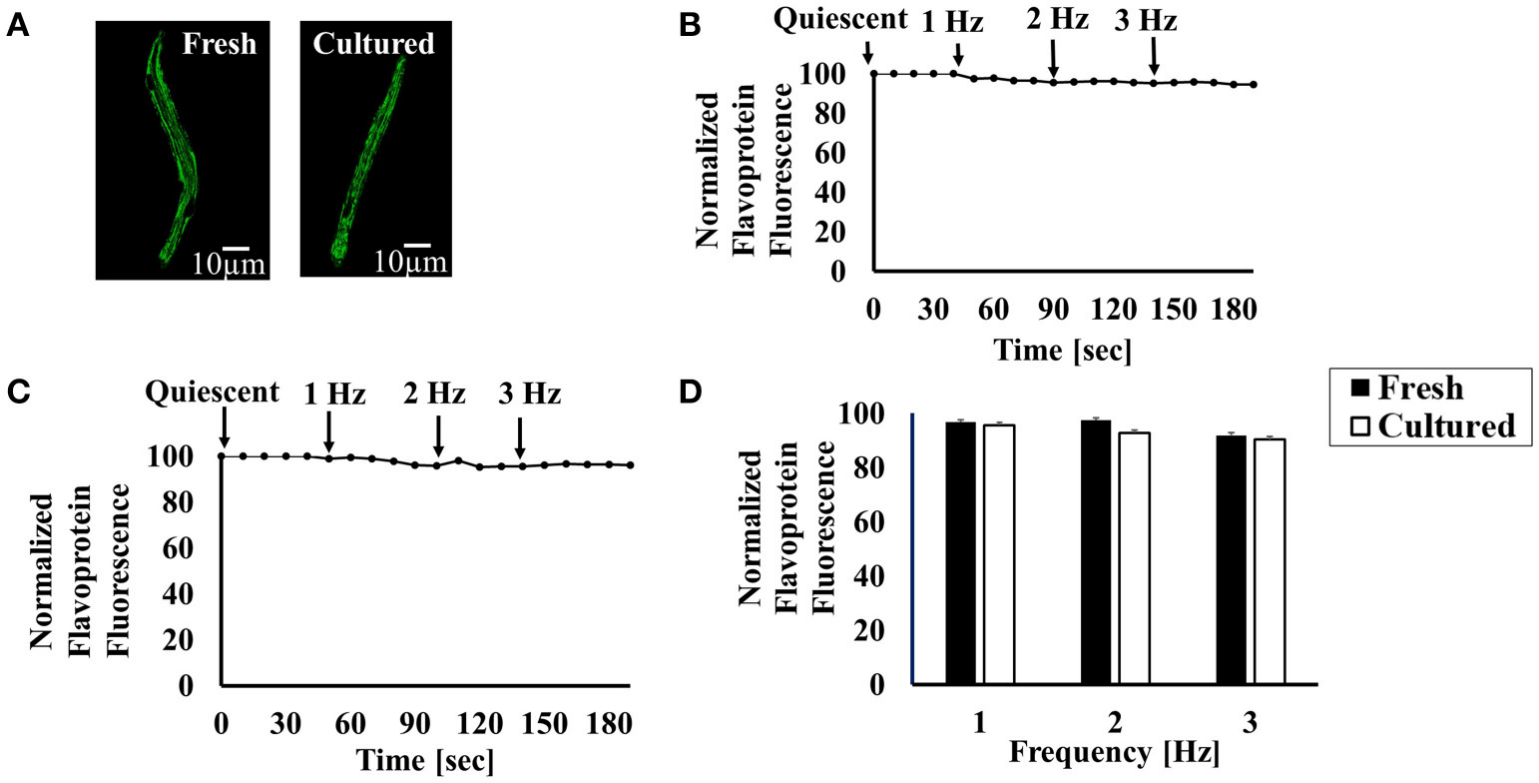
Bioenergetic characteristics of cultured cells. **(A)** Representative examples of fresh and cultured atrial cells loaded with TMRM. Representative examples of **(B)** fresh and **(C)** cultured atrial cell flavoprotein autofluorescence in response to increased electrical stimulation rate (from 1 to 3 Hz). **(D)** Average flavoprotein autofluorescence in response to increased electrical stimulation rate (from 1 to 3 Hz) (*n* = 32 fresh cells from 7 rabbits; *n* = 53 cultured cells from 7 rabbits).

As normal fresh heart cells have the ability to maintain ATP supply-to-demand matching in response to changes in demand (Covian and Balaban, 2012), we determined whether cultured atrial cells also possess this ability. First, we examined mitochondrial density by loading fresh and cultured atrial cells with TMRM to visualize the mitochondrial membrane. Figure 7A shows mitochondrial density in both cultured and fresh atrial cells. Analysis of average intensity of TMRM per cell shows that this index does not change between fresh and cultured cells: 95 ± 7 (*n* = 20; cells were taken from 7 rabbits) for fresh cells vs. 121 ± 3 (*n* = 20; cells were taken from 7 rabbits), respectively (*p* = 0.09). Thus, mitochondrial density is similar in fresh and cultured cells. Note that we used TMRM to visualize the mitochondrial membrane potential and not to quantify its value.

Next, we measured flavoprotein autofluorescence as an index of energy supply in response to a change in the demand. By electrically stimulating the cells or increasing the stimulation frequency, the cell demand for ATP increases (Brandes and Bers, 1997; Levy et al., 2005). If ATP supply to demand matches, no change in flavoprotein autofluorescence should occur at steady state. If ATP supply cannot match the demand, the fluorescence should increase. Figure 7B shows a representative example of fresh cells where an increase in pacing frequency does not change the ATP supply-to-demand balance (i.e., flavoprotein fluorescence remains constant). Figure 7C depicts an example of cultured cells exhibiting a similar behavior. Thus, on average (Figure 7D), both fresh and cultured cells maintained their ATP supply-to-demand matching in response to increased demand (increased pacing frequency).

## Discussion

In this study we developed an improved protocol for culturing adult rabbit atrial cells that preserve their bioelectric, biophysical and bioenergetic characteristics. Our method maintains the cells' ability to be externally paced in the physiological range of frequencies (1–3 Hz), their ability to generate, in response to electrical pacing, Ca^2+^ transients and local Ca^2+^ spark release similarly to fresh cells, and their ability to maintain ATP supply-to-demand matching in response to increased demand. Importantly, we showed that exogenous proteins can be expressed at high levels in the cultured cells within 24 h via adenoviral gene transfer, with no significant changes in cell volume.

To date, several methods have been developed for culturing mammal atrial cells and, specifically, rabbit atrial cells. Whereas, in one approach, cells could not maintain their elongated shape (Gilliam et al., 1989), in others the shape could be maintained but the cells could not be electrically paced at a rate higher than 0.5 Hz (Rinne et al., 2010; Hohendanner et al., 2015). This frequency is far from the physiological range and, therefore, the bioelectric, biophysical and bioenergetic parameters of these cultured cells do not accurately represent healthy atrial cells. To the best of our knowledge, this is the first method that enables the bioelectric, biophysical and bioenergetic characteristics of healthy cells to be preserved in culture.

BDM has previously been used for cell culture (Hall and Hausenloy, 2016). The authors found that BDM reduces spontaneous contractions per min and increases survival rate, but also reduces oxygen consumption. Similarly, Kabaeva et al. have found that BDM increases survival rate (Kabaeva et al., 2008). Finally, Kivisto et al. have found that BDM maintains cell shape, increases survival rate, and also maintains AP parameters (Kivistö et al., 1995). However, these experiments differ from our approach here in several important ways: (i) All of them were performed on ventricular cells and never on atrial cells. (ii) The ability of these cells to be stimulated at a high frequency rate was never tested. (iii) All these experiments were performed on mice or rats, inferior to rabbits in terms of their ability to serve as a model of human heart function (Bers, 2002). (iv) These experiments measured neither the ability cells to maintain Ca^2+^ transient and energetic balance in the entire physiological range of electrical pacing rates, nor their ability to maintain AP firing at physiological electrical pacing rates.

By nature, BDM prevents cell contraction. However, BDM has other side effects that may contribute to its preservation of atrial cell function: (i) altering of L-type Ca^2+^ and potassium channels; (ii) reduced Ca^2+^ releases (Gwathmey et al., 1991); (iii) reduced oxygen consumption (Hall and Hausenloy, 2016); (iv) reduced arrhythmogenic events (Lou et al., 2012); (v) reduced spontaneous contractions per min (Hall and Hausenloy, 2016). The effect on ionic channels and Ca^2+^ releases may reduce the ATP needed to maintain the AP and SR Ca^2+^ homeostasis and thus reduce oxygen consumption. Note that cells' ability to maintain AP and Ca^2+^ characteristics in culture similar to those of fresh cells implies that the BDM can be easily washed from the culture medium. The reduction in arrhythmogenic events and in spontaneous contraction imply that there will be less Ca^2+^ overload, and thus cell viability may increase.

**Figure 8:**
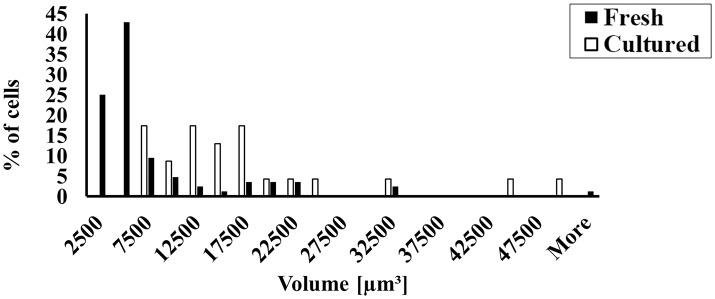
Maintaining sinoatrial node cultured cell volume and shape. Fresh and cultured cell volume distribution (*n* = 23 fresh cells from 4 rabbits); (*n* = 84 cultured cells from 4 rabbits).

Although, the right atrium is attached to the sinoatrial node, our culture method is not suitable for rabbit sinoatrial node cells. Figure 8 shows that although adding BDM to the culture medium results in partially maintained sinoatrial node cell volume, the cells' ability to beat spontaneously is not maintained. Several indications suggest that the isolated cells are purely atrial, and are not mixed with sinoatrial node cells: (i) The cells can be externally paced, whereas sinoatrial node cells cannot be paced for more that few beats (Yaniv et al., 2013). (ii) The morphology of atrial and sinoatrial tissues is completely different (Lyashkov et al., 2007). (iii) We removed all the transparent tissue (i.e., the sinoatrial node tissue) before isolating the atria. (iv) We ensured that the fresh isolated atrial cells did not beat spontaneously after isolation (in contrast to sinoatrial cells).

### Limitation

Note that some labs can maintain their cells in culture for more than 24 h (Gilliam et al., 1989; Rinne et al., 2010; Hohendanner et al., 2015). However, because our goal was to infect the cell with a virus, 24 h were sufficient. Thus, we did not extend the culture period to more than 24 h. Because multiple infection by some viruses might be required in the future, this method will have to be tested for a longer period.

We electrically paced the cells at 3 Hz, which is close to the physiological rate of isolated rabbit sinoatrial node cells. It is harder to maintain the beating rate even of fresh cells above this rate without isoproterenol or a higher Ca^2+^ load.

Note that although we found no change in local Ca^2+^ releases and the structure of the Ca^2+^ proteins, other compensatory mechanisms (such as the SR Ca^2+^ load or RyR Ca^2+^ sensitivity) may lead to preservation of biochemical and biophysical properties in cultured cells.

The representative figures and the bar graph (Figure 1) show that the rectangular shape of the cell is maintained. Because the cell is attached to the dish, some rounding can be observed. Similar rounding appeared with different media. Because no changes in structure and function of the cultured cell were observed, one can conclude that this effect is minor.

### Summary

In summary, this new method for culturing rabbit atrial cells provides proper sustenance of various atrial myocyte properties. This method has the potential to further our understanding of energetic and biochemical regulation in the atria, which can lead to novel therapies involving the adaptation of biophysical and bioenergetic signaling in the atria, with the goal of eliminating AF events.

## Author contributions

YY conceived the project. NKP and RB planned the experiments. NKP, SS, RB, BE, and YS executed the experiments. NKP and LAG analyzed the data. MD designed the analysis programs. YY and NKP wrote the paper with input from all authors (SS, RB, BE, YS, LAG, MD and OB). YY supervised all aspects of the work.

### Conflict of interest statement

The authors declare that the research was conducted in the absence of any commercial or financial relationships that could be construed as a potential conflict of interest.
